# 国际多学科肺癌组织病理新分类解读

**DOI:** 10.3779/j.issn.1009-3419.2013.06.01

**Published:** 2013-06-20

**Authors:** 冬梅 林

**Affiliations:** 100021 北京，中国医学科学院，北京协和医学院肿瘤研究所肿瘤医院病理科

肺癌组织学分类主要有非小细胞癌和小细胞癌两大类。1999年以及2004年两版的国际卫生组织（World Health Organization, WHO）/国际肺癌研究协会（International Association for the Study of Lung Cancer, IASLC）肺癌组织学分类之间无太大变化^[1]^。随着肺癌分子病理及靶向治疗研究的进展，2011年IASLC、美国胸科协会（American Thoracic Society, ATS）和欧洲呼吸协会（European Respiratory Society, ERS）等国际肺癌研究机构主要对其中肺腺癌分类进行了较为详细的修订以适应目前临床诊治的需求^[2]^。本文针对国际多学科肺癌组织病理新分类中的概念更新及相关诊断要点，并结合临床治疗、肿瘤预后或研究进展等内涵进行解读说明。

## 1 癌前病变

肺癌癌前病变主要有三种：鳞状上皮不典型增生和原位癌、非典型性腺瘤样增生和原位癌、弥漫性特发性肺神经内分泌细胞增生，分别对应的是鳞癌癌前病变、腺癌癌前病变以及类癌癌前病变。到目前为止，对于小细胞癌的癌前病变，尚未见任何报道及相关线索。

### 1.1 鳞状上皮不典型增生（squamous dysplasia, SD）和原位癌（carcinoma *in situ*, CIS）

1999年版WHO肺癌分类已经将鳞状上皮原位癌归入癌前病变，因此鳞状上皮癌前病变包括鳞状上皮非典型增生和原位癌。由于原位癌中的异型细胞没有突破上皮基底膜，尚未发展到浸润和转移的程度，从严格意义上来讲并不属于癌。因此，对于原位癌切忌按照真正意义上的癌过度治疗。近年来，为了与真正意义上的癌鉴别，WHO在许多器官组织学分类中已经废弃原位癌的概念，而代替为高级别上皮内瘤变。高级别上皮内瘤变的概念已经在许多器官肿瘤诊断中被广泛应用。所以，在规范病理诊断术语中，轻度、中度以及重度不典型增生等癌前病变的概念将逐渐被淘汰而取代为低级别和高级别上皮内瘤变等相关名称。

### 1.2 非典型性腺瘤样增生（atypical adenomatous hyperplasia, AAH）和原位癌

AAH是目前比较明确的肺腺癌癌前病变，在肺癌外科切除标本中，AAH占到2.4%-5.7%。病变结节往往边界不清，直径通常 < 0.5 cm，少数情况下直径可以超过0.5 cm，偶尔，也有可能达到1.0 cm。随着影像学技术的发展，体积较小的AAH病变逐渐被发现，影像以“毛沙状”改变为特点。镜下组织学表现为肺泡结构完好，肺泡上皮增生呈一致的立方形或矮柱状，有轻度非典型性，核仁缺乏或模糊。从细胞形态到组织结构有时难以与既往所谓细支气管肺泡癌（bronchioloalveolar carcinoma, BAC）鉴别，并可出现与BAC成分的过渡移行状态^[1, 3, 4]^。AAH和所谓的BAC或肺腺癌存在一些相似的分子异常改变，包括克隆性、*KRAS*突变及多态性、*EGFR*突变、*p53*表达及杂合性丢失和甲基化等^[5, 6]^。

### 1.3 原位腺癌（adenocarcinoma *in situ*, AIS）

2011年多学科肺腺癌组织学分类对肺腺癌癌前病变概念做了进一步延伸与调整，建议废弃BAC的名称，将病灶≤3 cm，瘤细胞局限于正常肺泡结构内（贴壁式生长），并且缺乏间质、脉管或肺膜浸润的病变定义为AIS。AIS主要是1999年和2004年版WHO分类中病灶≤3 cm的BAC，分为粘液型和非粘液型。AIS以非粘液型多见，由Ⅱ型肺泡上皮和/或Clara细胞组成。粘液型AIS比较罕见，几乎均由高柱状细胞组成，其细胞核位于基底，胞浆富于粘液，有时类似于杯状细胞。AIS细胞核异型性不明显，常见肺泡间隔增宽伴纤维化，尤其多见于非粘液型。AIS手术切除无病生存率为100%。

### 1.4 弥漫性特发性肺神经内分泌细胞增生（diffuse idiopathic pulmonary neuroendocrine cell hyperplasia, DIPNECH）

该病变逐渐证明是一种少见的与肺类癌和不典型类癌有关的癌前病理改变。DIPNECH主要位于支气管壁内或周围，体积 < 0.5 cm。主要表现为散在瘤样增生的细胞结节（神经内分泌小体）或岛状细胞团，细胞大小一致、核深染、核仁不明显、染色质细腻，核分裂像罕见且无坏死。当该增生区域 > 0.5 cm时即可诊断为类癌。作为最常见的肺神经内分泌癌类型，小细胞癌很可能直接发生于弥散神经内分泌细胞上皮，而不经过复杂的癌变病理过程。

## 2 肺癌

### 2.1 腺癌（adenocarcinoma, AC）

**腺癌组织亚型**

**取消BAC类型肺癌**  1999年版国际肺癌分类对BAC类型的诊断制定了明确而严格的标准：当全部的肿瘤成分均表现为沿肺泡壁结构生长（lepidic pattern），且无间质浸润、胸膜侵犯及脉管瘤栓，因此BAC类型只有在手术切除的标本中才得以诊断，并且从理论上讲应该处在原位癌阶段，这也就不难解释BAC类型的肺癌患者5年甚至10生存率可达到100%，因此细支气管肺泡癌有别于其它类型的肺腺癌，而且有关临床Ⅲ期或Ⅳ期BAC、肺内转移性BAC等说法应该是错误的。2011年IASLC等机构出台的肺腺癌新分类建议废除BAC类型，将大部分BAC明确归入原位腺癌的范畴，具体如下：

**Table:** 

**腺癌癌前病变**
**AAH**
**原位腺癌（既往≤3 cm的BAC）:包括非粘液性、粘液性、混合粘液与非粘液性**
**微小浸润性腺癌：≤3 cm的BAC，浸润范围≤5 mm**
**浸润性腺癌：**
**附壁结构为主型（lepidic predominant）：非粘液性BAC，浸润范围 > 5 mm**
**腺泡为主型**
**乳头为主型**
**微乳头为主型**
**实体伴粘液分泌型**
**浸润癌变异型**
**浸润性粘液腺癌：既往粘液性BAC**
**胶样癌**
**胎儿性腺癌**
**肠型腺癌**

**微小浸润性腺癌（minimally invasive adenocarcinoma, MIA）**   作为肺腺癌混合类型中最为常见的成分之一，BAC成分常常出现在癌灶的外围部分。多年来国际肺癌病理分类并未包含对其含量比例的临床意义及指导治疗等有重要价值的信息。不断有研究^[7-9]^显示肺腺癌BAC成分含量有一定的预后意义，故新分类中单独列出微小浸润腺癌的类型。MIA定义为≤3 cm以贴壁式生长为主的单发腺癌，包括非粘液型及粘液型，以非粘液型多见。界限清楚，浸润间质最大径≤5 mm。肺内多灶发生的腺癌也可适用于MIA的诊断。MIA浸润成分判定如下：①贴壁式生长之外的组织学亚型（即腺泡型、乳头型、微乳头型和/或实性型），或②肿瘤细胞浸润纤维母细胞性间质。如果肿瘤侵犯脉管或胸膜或者有坏死，则不能诊断为MIA。MIA病灶经完整切除后，总体5年生存率为100%。

**浸润性腺癌亚型（subtype of invasive adenocarcinoma）**  肺腺癌是一类异质性非常大的肺癌亚型，以往分类通常分为以下五种类型：腺泡型、乳头型、细支气管肺泡癌（BAC）、伴有粘液分泌的实体性腺癌、混合型，实际上80%以上肺腺癌为前四种类型（至少两种以上）的混合型，而这种混合成分种类及含量多少在很大程度上影响了病理诊断的差异性，同时也增加了患者病理类型与治疗或预后判断对比的评价难度。IASLC/ATS/ERS多学科国际协作新分类提出取消2004年版WHO分类中“混合型腺癌”亚型，可以改为半定量方式提示各亚型含量，以优势成分命名并提供各类型含量的百分比^[2]^。至于具体亚型仍保留原分类中的腺泡型、乳头型以及粘液分泌的实体性腺癌三个亚型，增加了附壁为主型[（lepidic predominant, LP）既往≥3 cm的BAC类型腺癌，或≤3 cm的BAC类型腺癌但浸润范围≥5 mm]及微乳头型。

注意如果肿瘤形态类似MIA，但出现侵犯淋巴管/血管/胸膜，或出现坏死时，应诊断LP，因此准确判断局部胸膜是否受累显得尤为重要。因为原来BAC类型的腺癌沿肺泡壁达胸膜下时，病理医师常常认为累及脏层胸膜。其实大多数情况下胸膜没有真正受侵，国际肺癌组织建议行弹力纤维染色观察胸膜是否存在弹力纤维断裂来确定胸膜是否受累。另外LP类型中的附壁结构特指非粘液性成分，因此不要出现以附壁结构为主的浸润性粘液性肺腺的诊断术语。至于微乳头型腺癌类型则是与临床关系比较密切，即使少到只有5%的含量，它的出现预示着肿瘤有较强的侵袭性和转移能力。目前所有研究^[10, 11]^均表明该亚型为主的早期肺癌预后不良，但在肿瘤晚期由于受临床分期的影响，该结构不是独立的预后因素。

**浸润性腺癌变异型**（variation of invasive adenocarcinoma）  分为四个变异型：浸润性粘液腺癌、胶样癌、胎儿型腺癌、肠型腺癌，前三个类型保留了原分类类型，只是浸润性粘液腺癌原分类称之为粘液性囊腺癌，实际病理改变为 > 3 cm，或浸润范围 > 5 mm的粘液性BAC类型；而肠型腺癌是本次新分类中增加的一个亚型，指形态上类似肠道腺癌即高柱状管状或筛状腺癌，且可以表达CDX-2蛋白，与肠癌肺转移难以鉴别，TTF-1、Napsin-A的阳性表达可以将二者区分。

此新分类在病理形态与预后关系方面进一步细化，提出了和临床治疗、判断预后密切相关的亚型分类，与原分类相比显示其良好临床应用价值^[12, 13]^。病理医师诊断时应严格掌握并按照国际标准进行分类以尽可能保证结果的一致性，但也确实存在一些目前尚难以解决的问题，比如肿瘤间质纤维化不甚明显时，如何判断附壁结构有无浸润？因为还没有类似检测乳腺导管内癌或前列腺腺体基底膜是否完整的相关标记物来判断附壁结构的完整或受侵状态，诊断时只能依靠病理医师的经验或主观判断，势必会造成诊断亚型的分歧；另外冰冻诊断时由于取材有限，如何恰当提示肿瘤的类型，这种情况下可以借鉴小标本的处理原则，提示肿瘤主要表现为附壁型结构，需石蜡标本充分取材观察后明确亚型。

### 2.2 鳞状细胞癌（squamous cell carcinoma, SCC）

肺鳞癌是非小细胞肺癌主要组织学类型之一，多年来无形态学亚型变化。主要变异型有：乳头状细胞型、透明细胞型、小细胞型、基底细胞型。其中小细胞型鳞癌需要与小细胞癌鉴别，有时仅凭形态对两者进行诊断鉴别非常困难，但由于两者的治疗方案差别较大，所以在活检诊断时，应尽量将二者区分，可以借助免疫组织化学染色技术鉴别诊断，但有些情况下不能保证可以将其完全明确区分，比如肿瘤成分太少、免疫组化检测结果有交叉或不满意等情况^[14-16]^。

1999年版到2004年版WHO明确规定在小活检标本，当分化较差很难区分低分化鳞癌和低分化腺癌时，可以笼统归为非小细胞肺癌（non-small cell lung cancer no specific, NSCLC NOS）。随着肺癌靶向治疗研究的不断进展，非小细胞肺癌的诊断名称已经不能满足临床的需要。2011年新分类则要求在小活检标本中，应借助于免疫组化检测尽可能提示腺癌或鳞状细胞癌类型^[2]^，只有当形态学或免疫表型结果不典型或两者有矛盾时，才可使用NSCLC NOS的诊断。病理报告中不要使用非鳞状细胞癌的诊断名词，该名词是仅限于临床医师为决定治疗方案所使用的一个术语。

对于临床医师而言应当了解病理诊断借助免疫组织化学检测免疫表型对肿瘤亚型分类有很大帮助，但不能保证100%可以明确分型。在诊断不肯定、类型不明确或诊断与临床有不符之处时应注意与病理医师之间的沟通和交流。

### 2.3 大细胞癌（large cell carcinoma, LCC）

大细胞癌为分化差的非小细胞癌一种独立类型，LCC占到所有肺癌的9%，在光镜下看不到任何腺或鳞的分化特征，只是电镜下有腺或鳞的分化迹象，所以该类型肺癌也只有在手术切除的标本中才能诊断，且不能借助于免疫组化来辅助判断LCC。但由于对该类型肿瘤诊断标准掌握的不严格，国内LCC的诊断并未达到9%的比例，而是将有些病例错误地划入了低分化腺鳞癌的范畴。

### 2.4 腺鳞癌（adenosquamous cell carcinoma）

腺鳞癌只占据所有肺癌的0.6%-2.3%，属少见肺癌类型。当癌组织中同时含有腺癌和鳞状细胞癌成分，且每种成分至少占10%以上时才可诊断为腺鳞癌，故该类型肺癌也是只能出现在手术切除的大标本病理报告中。诊断时切忌将似有腺或鳞状分化、但分化证据又不足的大细胞癌亚型误归入低分化腺鳞癌类型。

### 2.5 肉瘤样癌（sarcomatoid carcinoma）

肉瘤样癌为一类分化差的非小细胞癌，其亚类包括：多型癌、梭形细胞癌、巨细胞癌、癌肉瘤、肺母细胞瘤。其中多型癌的定义为任何一种非小细胞癌类型（腺癌、鳞癌、大细胞癌）混合肉瘤样成分如梭形细胞或巨细胞成分等；肺母细胞瘤好发于成人，由胚胎性间质和上皮成分组成。

### 2.6 神经内分泌癌（neuroendocrine carcinoma）

按照分化程度，神经内分泌癌分为高、中、低分化，具体对应的肿瘤类型为类癌、不典型类癌、小细胞癌/大细胞神经内分泌癌，对各类型的病理诊断均有明确指标要求。从组织发生、分子异常改变、组织形态学、免疫表现检测、临床治疗及预后等方面，小细胞癌与大细胞神经内分泌癌有很大的相似性。

#### 2.6.1 小细胞癌

小细胞癌的演变很复杂，目前只保留小细胞癌和复合型小细胞癌两个亚型，后者亚型是指小细胞癌混合非小细胞癌成分。该亚型提示临床其治疗效果可能不如单纯小细胞癌敏感。

#### 2.6.2 大细胞癌神经内分泌癌

与小细胞癌相似，有大细胞神经内分泌癌和复合型大细胞癌神经内分泌癌两个亚型。与小细胞癌的主要区别在于瘤细胞大小和形态，大小应大于3个正常淋巴细胞体积，形态显示瘤胞浆丰富，更具有上皮分化特征，核仁明显是其主要病理特点。同一肿瘤中也可出现大细胞神经内分泌癌与小细胞癌的混合成分，目前WHO分类认为此时可看作小细胞癌的亚型之一。

#### 2.6.3 类癌和不典型类癌

前者为低度恶性而后者恶性度稍高。两者之间的区别在于每10个高倍视野2个核分裂像为界，另外，小灶坏死的有无也是其鉴别诊断指标之一。与类癌相比，不典型类癌常发生于外周，转移率增加，预后相对较差。目前研究结果^[17]^表明10%-15%的典型类癌和40%-50%的不典型类癌发生区域淋巴结转移和5%-20%的远处器官，如骨或肝脏等的转移。类癌和其它肺癌不同，该肿瘤与吸烟无关，但在分子病理方面与其它类型的肺癌有许多相似之处。总的来说典型类癌和不典型类癌分子生物学方面的改变基本一致，不同的是3p和位于13p14的*RB*基因的等位基因缺失很少发生在类癌，但可发生于20%和40%不典型类癌中。类癌独有的改变特征是经常出现*MEN1*基因的突变但缺乏蛋白表达^[18]^，这种*MEN1*基因的等位缺失发生于11P13。与其它肺癌相比，除了*RASFF1A*（位于3p21.3）和*caspase-8*基因外，类癌中很少有基因发生甲基化。

### 2.7 涎腺源性癌（carcinoma of the salivary glands）

该类型肺癌来源于支气管粘膜腺体，与发生于涎腺内的相同类型癌一致，主要类型有腺样囊性癌、粘液表皮样癌、肌上皮癌、恶性多形性腺瘤等。

## 3 总结

总之，国际多学科肺腺癌新的组织学分类在提示肺腺癌预后及指导治疗方面提供了更加详细准确的病理信息。建议废除BAC类型，拓展了腺癌癌前的范畴，增加了MIA、LP亚型及肠型腺癌变异型；对于多种腺癌形态的病例，建议不再使用混合型腺癌类型，而代之于优势亚型为主并注明各成分含量的病理诊断报告模式；为满足晚期肺癌患者靶向治疗或个体化治疗的需求，建议在活检标本中尽可能区分腺癌、鳞状细胞癌亚型，必要时需进行免疫表型检测，尽量不使用NSCLC NOS的诊断名词。腺癌以外其它类型或亚型肺癌则仍保留2004年WHO肺肿瘤分类原则。
